# Efficacy and safety of sirolimus-eluting stents versus bare-metal stents in coronary artery disease patients with diabetes

**Published:** 2014

**Authors:** Juehua Jing

**Affiliations:** Department of Orthopaedics, Second Hospital of Anhui Medical University, Hefei, Anhui Province, People’s Republic of China

## Dear Sir

I read with great interest the recent article titled ‘Efficacy and safety of sirolimus-eluting stents versus bare-metal stents in coronary artery disease patients with diabetes: a metaanalysis’ by Qiao *et al.*, published online in the *Cardiovascular Journal of Africa*.^1^ I believe this is a well-conducted metaanalysis that compared the major cardiac events, target-lesion revascularisation, myocardial infarction and mortality rate in coronary arterial disease (CAD) patients with diabetes who were treated with sirolimus-eluting stents (SES) or bare-metal stent (BMS). However, there are some issues I would like to point out.

The electronic databases (PubMed, MEDLINE, EMBASE, Springer, Elsevier Science Direct, Cochrane Library and Google scholar) were systematically searched by the authors. However, they did not describe the search strategy for databases in detail, which plays an important role in systematic reviews. The manual searches were not clearly described. The lack of a manual search protocol may be considered a weakness of the meta-analysis.

The publication language in this meta-analysis was limited to English but the authors did not mention it in the discussion. Therefore, there may have been a language bias in their metaanalysis. I suggest that there be no language limitation for the included studies to reduce the bias.

The inclusion and exclusion criteria were not adequately described in this meta-analysis. I suggest that explicit inclusion and exclusion criteria be introduced in detail.

The publication bias in this meta-analysis was evaluated with Egger’s test and funnel plots. However, the number of studies was less than 10, and as far as I know, a funnel plot should be inspected visually to assess for publication bias in meta-analyses with at least 10 studies. Therefore, it was inappropriate.

Under the statistical analysis heading, the authors wrote ‘Pooled ORs were obtained using the Mantel-Haenszel method in a fixed-effect model, and the DerSimonian-Laid method in a random-effects model’. However, it is not appropriate to use the Mantel-Haenszel method in a random-effects model to pool the data for all forest plots, regardless of heterogeneity.

It is very important in meta-analyses to evaluate methodological quality of included studies. However, the authors did not provide any methodological quality assessment or detailed scores for each trial in this article.

In conclusion, I agree with the results of this meta-analysis. SES are safer and more effective than BMS in CAD patients with diabetes, as far as major cardiac events are concerned. To reach a definitive conclusion, however, more high-quality studies with larger sample sizes are needed.
